# Competing fragmentation processes of *O*-acetyl-substituted carboxylate anions subjected to collision-induced dissociation

**DOI:** 10.1177/14690667251346668

**Published:** 2025-05-29

**Authors:** J Stuart Grossert, Robert L White

**Affiliations:** Department of Chemistry, 98609 Dalhousie University, Halifax, Nova Scotia, Canada

**Keywords:** Acetyl esters, aspirin, carboxylic acids, collisional activation, DFT computations, ESI(-)-MS/MS, fragmentation mechanisms, ketene

## Abstract

Carboxylic acids containing an *O*-acetyl substituent were studied using tandem mass spectrometry (MS/MS). Decarboxylation was observed for deprotonated *O*-acetylmandelic acid, whereas deprotonated acetoxyacetic acid and acetylsalicylic acid fragmented by two competing pathways. In the lower energy process, the product ion was formed by intramolecular proton abstraction and subsequent neutral loss of ketene (CH_2_=C=O) from the *O*-acetyl group. At higher collision energies, nucleophilic displacement of the *O*-acetyl group by the carboxylate group of acetoxyacetate yielded acetate (CH_3_CO_2_^–^) as the more abundant product ion. The relative energetics computed for the reaction pathways of acetoxyacetate were consistent with the product ion spectra. Overall, the observation of both the loss of ketene and the formation of acetate ion are characteristic of an *O*-acetyl group in the precursor carboxylate ion undergoing collision-induced dissociation. The different fragmentation behavior exhibited by *O*-acetyl mandelate was attributed to the charge stabilizing properties of the phenyl substituent that facilitated decarboxylation. Thus, the fragmentation processes observed depended on the structures of the *O*-acetyl-substituted carboxylate ions and the associated intramolecular interactions.

## Introduction

Carboxylic acids are common organic substances that are also found in nature as free acids and as derivatives such as esters and amides. The acids are readily deprotonated by electrospray ionization (ESI) to form [M–H]^–^ (carboxylate) ions. When subjected to collision-induced dissociation (CID) using tandem mass spectrometry (MS/MS), the carboxylate ions undergo fragmentation that is influenced by structural aspects such as the presence and proximity of another functional group.

On collisional activation, carboxylate ions may fragment by decarboxylation.^1–6^ However, other fragmentation processes have been characterized for carboxylate ions with particular structural features. Thus, carboxylate ions are known to react by decarboxylative elimination^7^ and intramolecular nucleophilic displacement,^8,9^ as well as by processes that require proton abstraction, for example McLafferty-type rearrangement^10^ and formation of the hydroxycarbonyl anion.^11,12^ Another process indicated by a mass transition of 42 u was used in the multiple reaction monitoring technique for the determination of deprotonated acetylsalicylic acid (aspirin),^13–17^ an aryl carboxylate ion. This uncommon neutral loss of C_2_H_2_O was determined by accurate mass measurements and attributed to the loss of ketene (CH_2_=C=O) from the *O*-acetyl group.^18^ Although the loss of ketene from deprotonated, *N*-acetyl amino acids has been mentioned,^19^ recent studies have documented ketene formation as a prominent fragmentation reaction of several protonated *N*-acetyl amino acids^19^ and protonated β-amino acids.^20^

**Figure 1.**
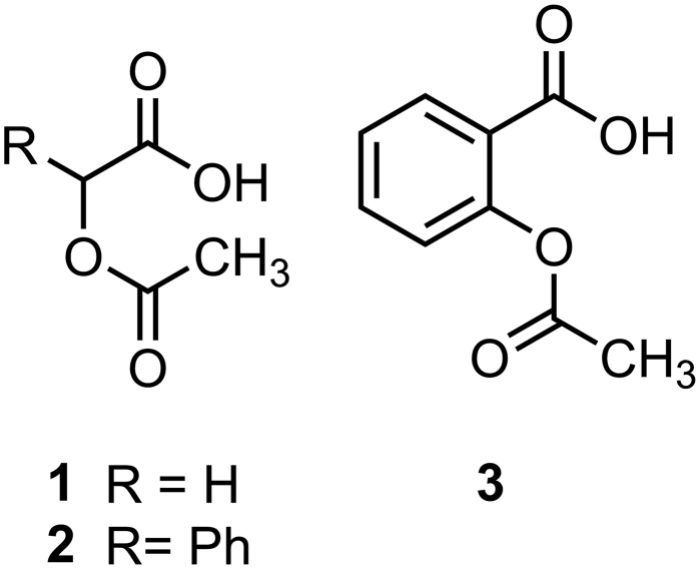
Structures of *O*-acetyl carboxylic acids. The corresponding ions formed by deprotonation are designated as **1a**, **2a**, and **3a**, respectively.

In the current investigation, the three carboxylic acids containing *O*-acetyl esters (Figure 1) were chosen to study the effects on fragmentation behavior of structural changes, such as the addition of a phenyl substituent and the geometrical constraints resulting from the inclusion of an aromatic ring. The results of tandem mass spectrometry and computations revealed that intramolecular interactions between the *O*-acetyl and the carboxylate groups led to competing fragmentation processes of the acetyl group in deprotonated acetoxyacetic acid (*O*-acetylglycolic acid, **1a**) and acetylsalicylic acid (2-acetoxybenzoic acid, **3a**), but primarily decarboxylation was observed in the case of deprotonated *O*-acetylmandelic acid (2-acetoxy-2-phenylacetic acid, **2a**).

## Materials and methods

Acetoxyacetic acid, *O*-acetylmandelic acid and acetylsalicylic acid were obtained from Sigma-Aldrich (Oakville, ON, Canada); each was dissolved in aqueous methanol (1 mg mL^–1^, 1:1 v/v) and a portion (5–10 μL) was introduced into the mass spectrometers by flow injection (20 μL min^–1^; syringe pump).

Mass spectra were collected on Thermo-Finnigan LCQ-Duo ion-trap and Micromass (now Waters) Quattro triple quadrupole mass spectrometers. In the ion-trap mass spectrometer,^21^ the voltage on the electrospray needle, the capillary temperature, sheath gas flow rate, and CID isolation width were set at 4.0 kV, 200°C, 20 arbitrary units, and 1 u, respectively. Spectra were collected (typically 20–30 scans) using helium as the damping and collision gas, a source potential of 0–25 V, and processed using Xcalibur software. In the triple quadrupole mass spectrometer,^11^ the electrospray needle and the source temperature were set at 4.0 kV, and 90–100°C, respectively. The gas for the bath and nebulizer was nitrogen. Spectra collected (typically 20–30 scans) using argon as the collision gas at energies of 5–20 eV (laboratory frame) and a cone voltage of 10–30 V were processed using MassLynx software.

Assignments of fragment ion structures were based on comparison of *pseudo* MS^3^ spectra with literature spectra and by comparison with the documented fragmentations of ions with analogous structures. The structures were consistent with computational results, mechanistic reasoning, and the structure of the precursor ions.

As reported for previous computations,^7^ geometry optimizations and frequency calculations were performed using the B3LYP/6–31++G(2d,p) functional, and the MP2/6-311++G(2d, p)//B3LYP/6-31 ++G(2d, p) composite level of theory was used to determine zero-point-corrected free energies, which are reported in kJ mol^‒1^. Cartesian coordinates for the optimized structures are given in the supplemental material.

## Results and discussion

### Acetoxyacetate, 1a

**Figure 2.**
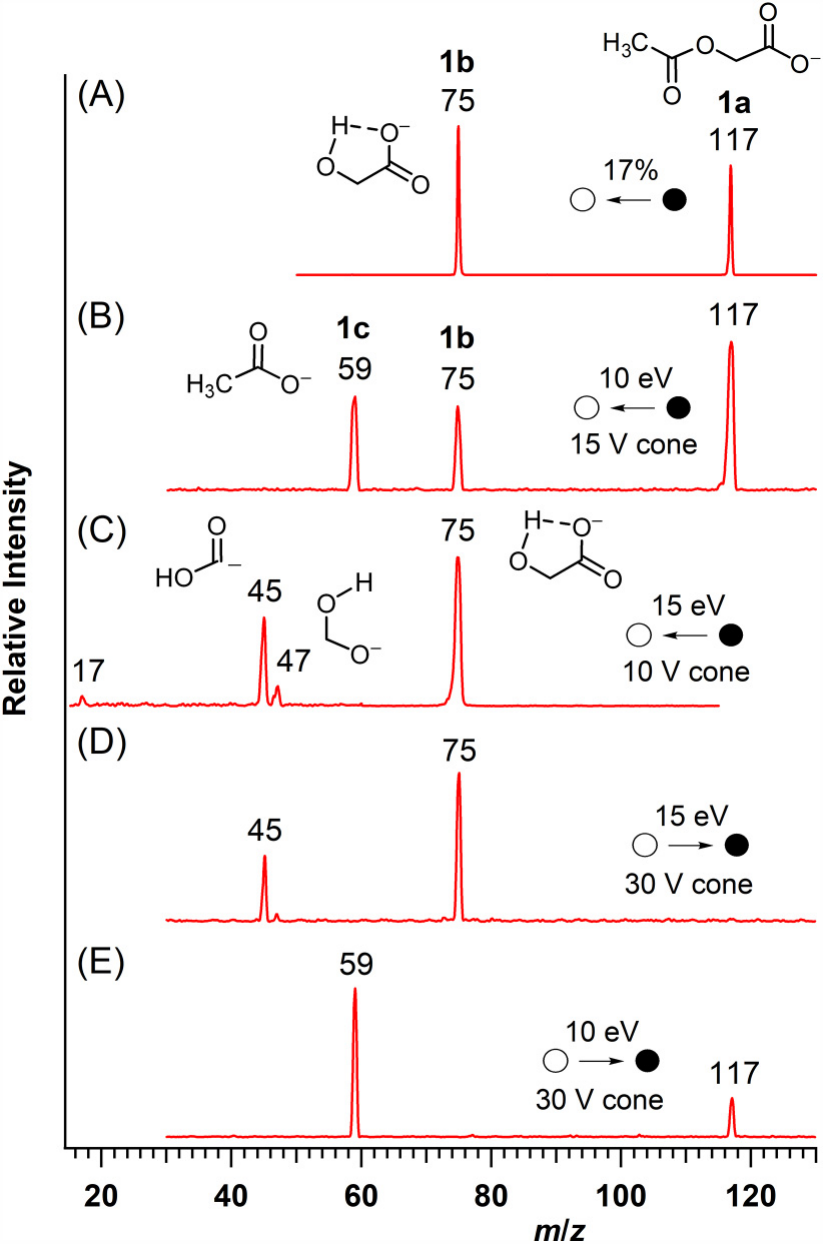
Mass spectra collected for acetoxyacetate (**1a**). A. MS/MS of acetoxyacetate; B. MS/MS of acetoxyacetate; C. Pseudo MS^3^ of the ion at *m*/*z* 75; D. Precursor-ion spectrum of *m*/*z* 45; and E. Precursor-ion spectrum of *m*/*z* 59. Spectrum A was collected on the ion trap spectrometer, whereas spectra B–E were collected on the triple quadrupole spectrometer.

The tandem mass spectra of acetoxyacetate (**1a**, *m*/*z* 117; Figure 2, spectra A and B) showed the formation of an abundant product ion at *m*/*z* 75 (**1b**). A second product ion (**1c***, m*/*z* 59) was also detected in the triple quadrupole mass spectrometer. When **1b** (*m*/*z* 75) was formed from acetoxyacetate in the source of the mass spectrometer and selected for CID, the resulting pseudo MS^3^ spectrum (Figure 2C) matched that of deprotonated glycolic acid (HOCH_2_CO_2_^–^).^11^ A precursor-ion scan (Figure 2D) confirmed the precursor–product relationship for the ions at *m*/*z* 75 and 45 (HO_2_C^–^). The identification of the ion at *m*/*z* 75 as glycolate defined the neutral loss of 42 u from acetoxyacetate as C_2_H_2_O, the molecular formula of ketene (CH_2_=C=O).

At a collision energy of 5 eV in the triple quadrupole mass spectrometer, glycolate (**1b**, *m*/*z* 75) was the most abundant product ion formed from acetoxyacetate (**1a**); however, at collision energies ≥10 eV (e.g. Figure 2B), the product ion at *m*/*z* 59 (**1c**) was more abundant. These observations, together with the observation of glycolate as the only product ion detected in the ion trap (Figure 2A) indicated that formation of the ion at *m*/*z* 59 required a higher input of energy. The pseudo MS^3^ spectrum (Figure 2C) and precursor-ion scans (Figure 2, Spectra D and E) demonstrated that product ion **1c** and glycolate (**1b**) were formed from acetoxyacetate (**1a**) by independent, competing processes.

**Figure 3.**
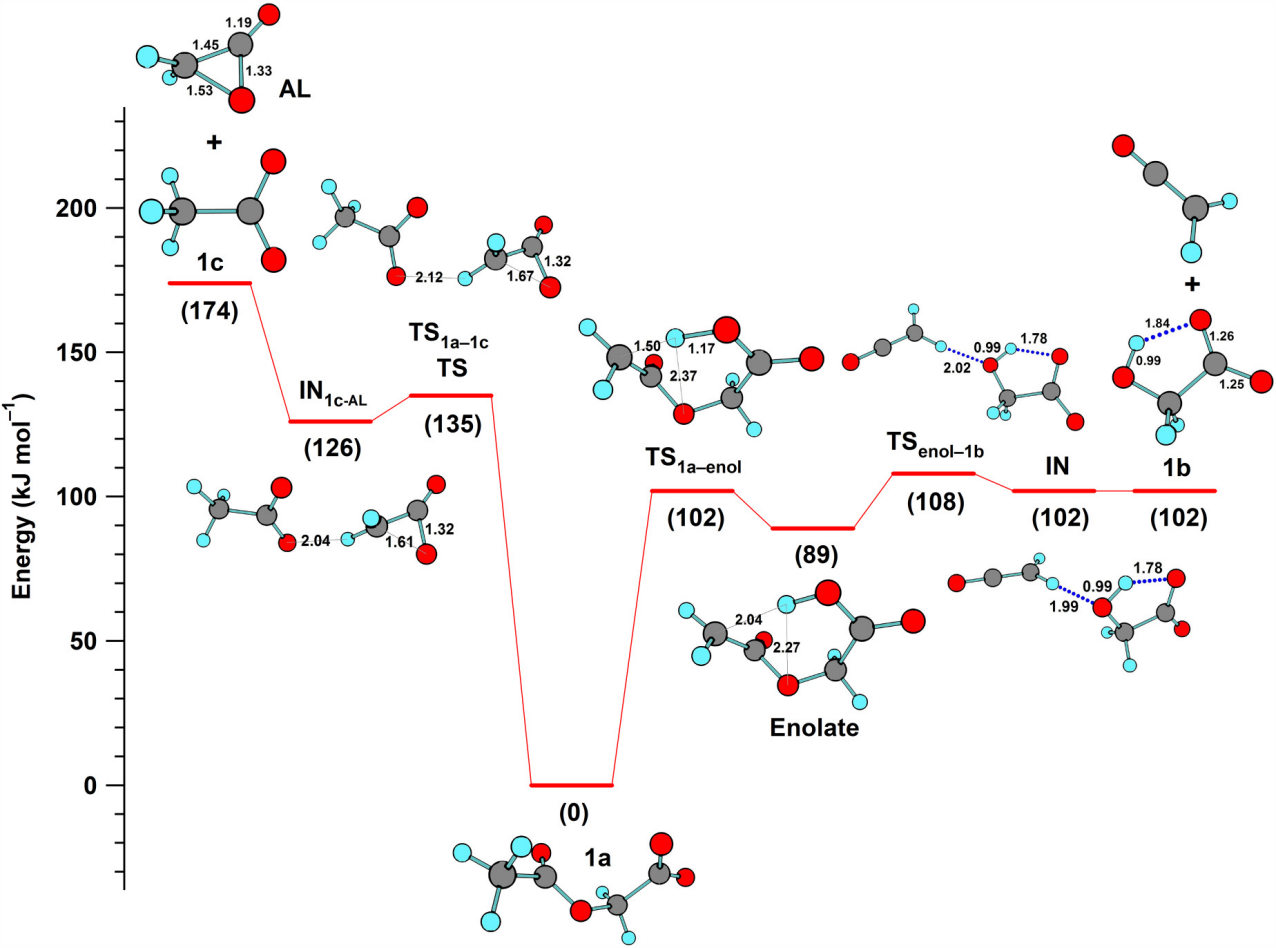
Potential energy profiles computed for the endergonic, competing fragmentation reactions of acetoxyacetate (**1a**): formation of glycolate (**1b**) and ketene (right-hand side); and formation of acetate (**1c**) and acetolactone (left-hand side).

**Scheme 1.**
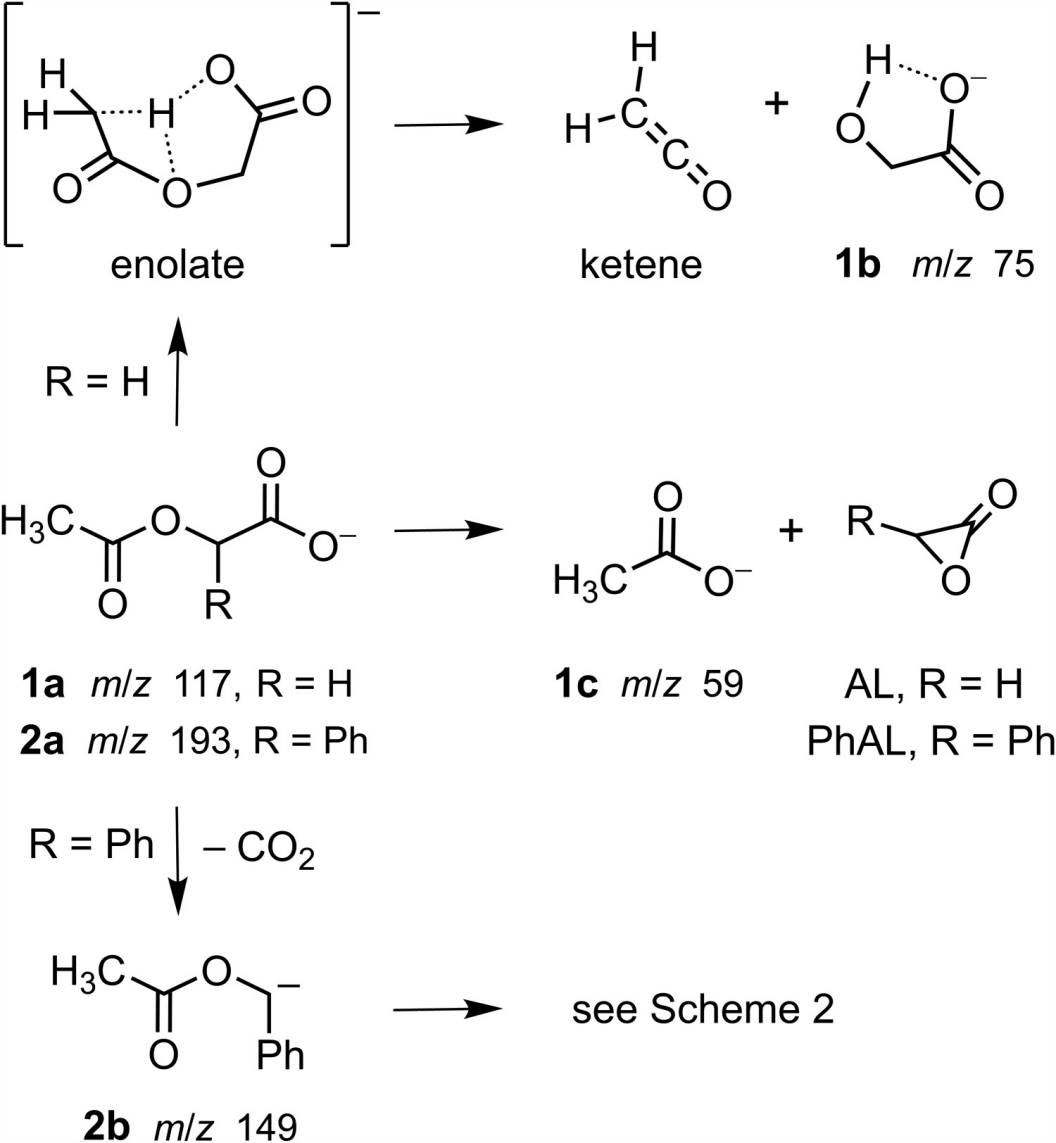
Competing proton abstraction and nucleophilic displacement reactions of acetoxyacetate (**1a**) and competing decarboxylation and nucleophilic displacement reactions of *O*-acetylmandelate (**2a**). The dotted lines in the enolate structure indicate hydrogen bonding to three centers.

AL: acetolactone; PhAL: phenylacetolactone.

The formation of products with masses corresponding to ketene and glycolate (**1b**) from acetoxyacetate (**1a**; Scheme 1) requires a rearrangement consistent with the intramolecular abstraction of a moderately acidic hydrogen on the methyl group by the ionized carboxyl group followed by bond cleavage. Ketene formation by the analogous C–C bond cleavage of deprotonated methyl ketones (R-CO-CH_2_^–^)^6^ is a close precedent for this process. The moderate difference between the gas-phase acidities of analogous compounds (i.e. methoxyacetic acid, Δ_r_G° ≈ 1405 kJ mol^–1^ versus methyl and ethyl acetate, Δ_r_G° ≈ 1530 kJ mol^–1^)^22^ indicates that enolate ion formation by abstraction of a proton from the acetyl group in acetoxyacetate (Scheme 1) is energetically reasonable. When the abstraction process was investigated computationally (Figure 3), the energies of acetoxyacetate and its enolate ion differed by only 89 kJ mol^–1^, and a modest barrier of 102 kJ/mol^–1^ was computed for proton abstraction via a seven-membered, cyclic transition structure (**TS_1a_**_–enol_). The latter was similar in magnitude to the barrier for the abstraction of a benzylic proton (97 kJ/mol^–1^) as the first step in the rearrangement of dihydrocinnamate prior to decarboxylation.^23^ Also, the barrier for proton transfer in acetoxyacetate was lower than that computed for the abstraction of a proton from oxygen in 2-hydroxycarboxylic acids (140–190 kJ/mol^–1^).^11^

Subsequent C–O bond cleavage of the enolate of acetoxyacetate was facilitated by the transfer of the abstracted proton to the ether oxygen of the acetyl group via a five-membered, cyclic transition structure (Figure 3, **TS**_enol–**1b**_). An input of only 19 kJ mol^–1^ was required to reach a loosely bound ion-neutral complex (IN) of ketene and glycolate (**1b**).

By analogy with the formation of phenoxide ion (PhO^–^ from phenoxyacetate (PhO–CH_2_CO_2_^–^)^24^ and halide ions from monohalogenated acetates,^8,9^ **1c** (*m*/*z* 59) was assigned as acetate ion (CH_3_CO_2_^–^), and its formation from the *O*-acetyl group (Scheme 1) was investigated computationally (Figure 3). The barrier for the intramolecular displacement of acetate by the carboxylate group in acetoxyacetate (135 kJ mol^–1^) was similar to that computed for the analogous displacement of phenoxide ion from phenoxyacetate (152 kJ mol^–1^).^24^ The neutral product for each process was acetolactone (AL), a three-membered lactone that also was formed by the displacement of halide ions from substituted acetate ions.^8,9^

The energetics computed for the fragmentation of acetoxyacetate (Figure 3) are consistent with the mass spectral observations. In the ion-trap mass spectrometer, where collisions with helium atoms add energy in small increments, only glycolate (**1b**), the product of the lower energy pathway, was observed (Figure 2A). In addition to the higher barrier computed for the initial step in the displacement pathway, additional input of energy (48 kJ mol^–1^) is needed to separate acetate and acetolactone from their ion-neutral complex IN**_1c−AL_**. This dissociation energy is greater than the small barrier (9 kJ mol^–1^) for the reverse reaction, and, when energy is added in small increments, return to the initial acetoxyacetate ion is favored. Consequently, only glycolate is observed upon CID in the ion trap. In the triple quadrupole mass spectrometer, collisions with argon atoms transfer energy greater than that computed for each process^25^ and two product ions are observed (Figure 2B). At low collision energy (5 eV, *E*_LAB_; 1.27 eV = 123 kJ mol^–1^, *E*_CM_), glycolate (**1b**), the product of the lower energy pathway, was more abundant, whereas the product of the higher energy pathway, acetate (**1c**), became the more abundant product ion at higher collision energies (e.g. Figure 2B: 10 eV, *E*_LAB_; 2.55 eV = 246 kJ mol^–1^, *E*_CM_).

### *O*-Acetylmandelate, 2a

**Figure 4.**
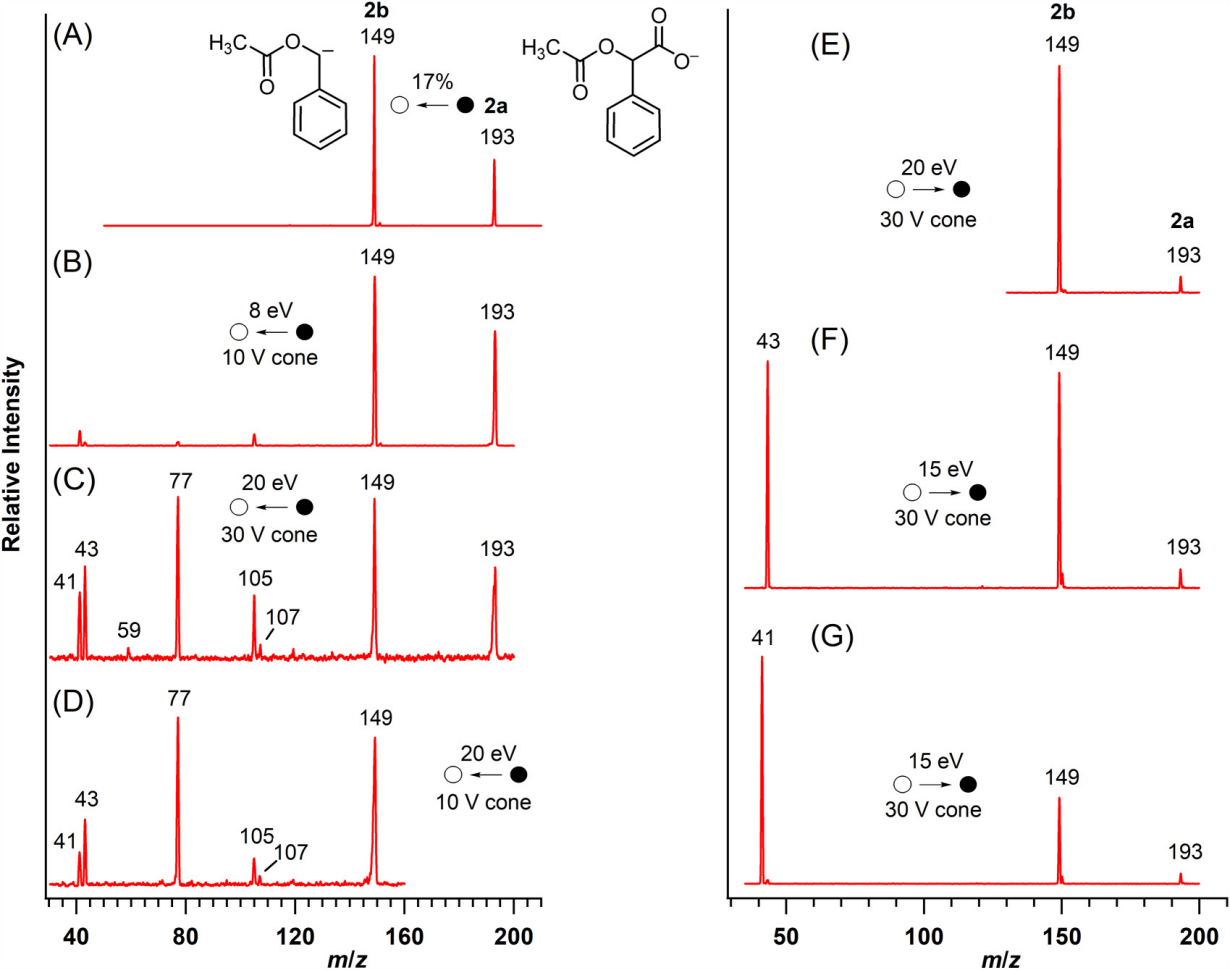
Mass spectra of *O*-acetylmandelate (**2a**). A. MS/MS of **2a**; B. MS/MS of **2a** at low collision energy; C. MS/MS of **2a** at high collision energy; D. Pseudo MS^3^ of the *m*/*z* 149 ion; precursor-ion spectra. E. *m*/*z* 149; F. *m*/*z* 43; and G. *m*/*z* 41. Spectrum A was collected on the ion-trap mass spectrometer and spectra B–H were collected on the triple quadrupole mass spectrometer.

In contrast to the fragmentation behavior of acetoxyacetate (vide supa), decarboxylation was the primary fragmentation process observed for *O*-acetylmandelate (**2a**; Figure 4, Spectra A and B; Scheme 1). At higher collision energy, the tandem mass spectrum (Figure 4C) showed the formation of six other product ions. Pseudo MS^3^ (Figure 4D) showed that five of these ions (*m*/*z* 107, 105, 77, 43 and 41), including two pairs of complementary ions at *m*/*z* 41/107 and *m*/*z* 43/105,^11,26^ were formed from the decarboxylated ion **2b** (*m*/*z* 149). Precursor-ion scans (Figure 4, Spectra E, F and G) linked the decarboxylated ion with the [M–H]^–^ ion and demonstrated that the ions at *m*/*z* 41 and 43 were formed from the decarboxylated ion **2b** by independent routes. The ion at *m*/*z* 59 (Figure 4C) was not detected in the pseudo MS^3^ spectrum of the decarboxylated ion **2b** (Figure 4D), suggesting direct formation from *O*-acetylmandelate (**2a**) by a competing nucleophilic displacement (Scheme 1).

**Scheme 2.**
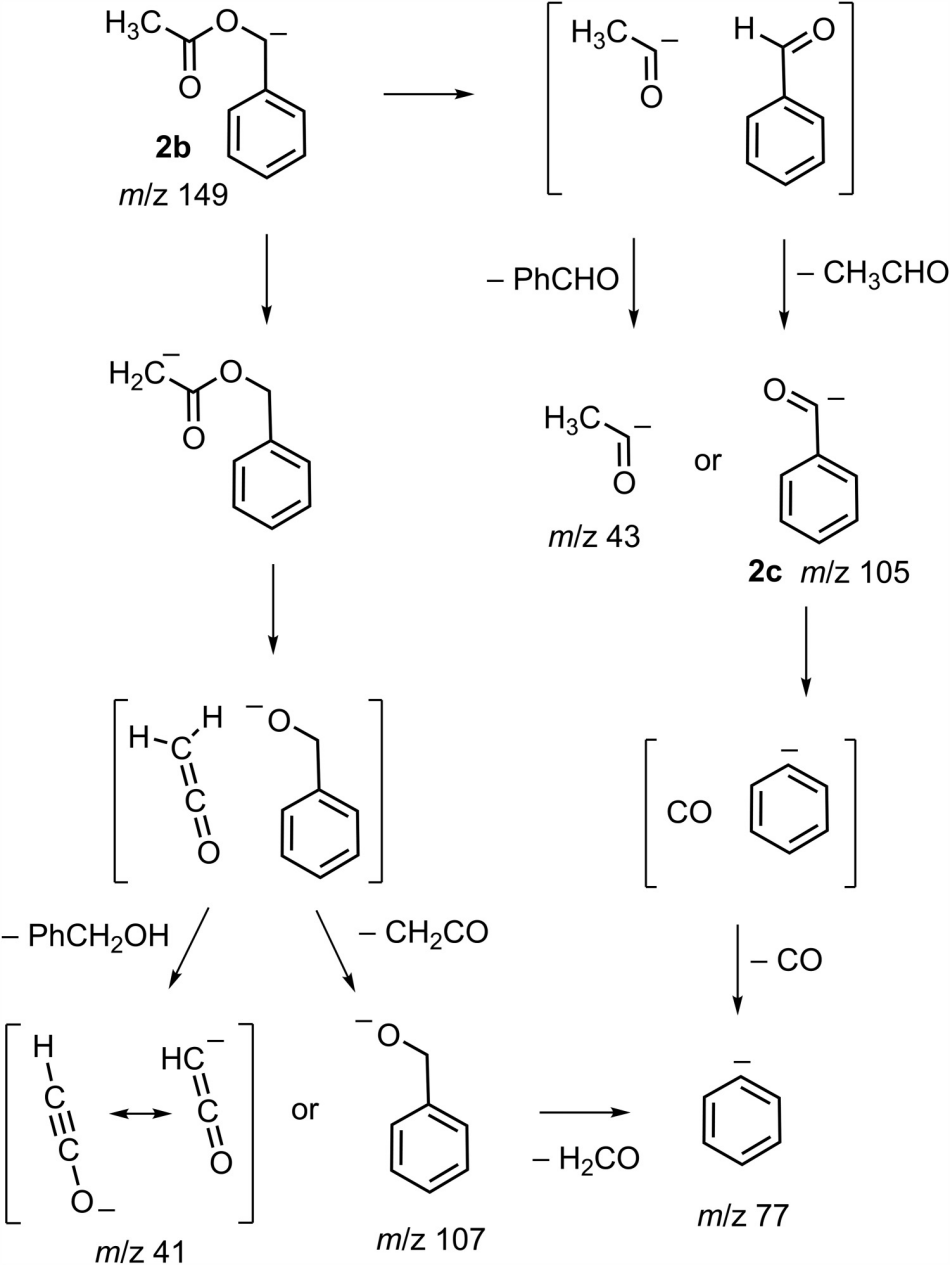
Plausible fragmentation reactions of the ion **2b** formed by decarboxylation of *O-*acetylmandelate (**2a**) upon CID (Scheme 1). Note that **2b** undergoes two competing fragmentation reactions each generating an ion-neutral complex.

*O*-Acetylmandelate (**2a**) and acetoxyacetate (**1a**) are analogs differing by a phenyl substituent (Scheme 1). However, this single structural difference has a significant effect on the fragmentation behavior. The phenyl substituent facilitates the decarboxylation of *O*-acetylmandelate (**2a**; Figure 4, Spectra A and B) by effectively stabilizing the charge developed at the benzylic position upon the neutral loss of carbon dioxide (Scheme 1).^3,27^ The prominent decarboxylation of *O*-acetylmandelate is consistent with the low threshold energy measured for decarboxylation of the analogous ion phenylacetate.^6,28^ Also, the subsequent fragmentation processes of the benzylic ion **2b** yielding product ions at *m*/*z* 107, 105, 77, 43 and 41 (Figure 4D) are consistent with the structure assigned (Scheme 2).

Formation of five product ions detected at lower *m*/*z* (Figure 4D) is consistent with the fragmentation of **2b** by two competing processes (Scheme 2). In one process, intramolecular abstraction of a proton from the methyl group precedes bond cleavage generating ketene and deprotonated benzyl alcohol (*m*/*z* 107) upon dissociation of the ion-neutral complex. Alternatively, abstraction of a proton from ketene prior to dissociation results in the formation of the complementary ketenyl ion at *m*/*z* 41^5^ and benzyl alcohol. Note that the gas-phase acidity of ketene (Δ_r_G° ≈ 1497 kJ mol^–1^) is slightly higher than that of benzyl alcohol (Δ_r_G° ≈ 1520 kJ mol^–1^)^22^ and that the abundance of the ketenyl anion (an ynolate) at *m*/*z* 41 is greater than that of its complementary ion at *m*/*z* 107. Loss of formaldehyde from deprotonated benzyl alcohol is a potential route to phenide ion (*m*/*z* 77).^24,29^

In the competing fragmentation process of **2b**, bond dissociation gives an ion-neutral complex from which the product ion at *m*/*z* 43 is generated by dissociation. Neutral acetaldehyde and the complementary acyl anion at *m*/*z* 105 (**2c**) are generated by proton abstraction prior to dissociation of the ion-neutral complex. Loss of carbon monoxide from the ion at *m*/*z* 105 yields phenide ion (*m*/*z* 77).^30^

Formation of the ion of low abundance at *m*/*z* 59 from *O*-acetylmandelate (**2a**) is consistent with the nucleophilic displacement of acetate by the carboxylate group with formation of a phenyl-substituted acetolactone. This is analogous to the formation of acetate from acetoxyacetate (**1a**; Figure 3). In both instances, acetate ion (*m*/*z* 59) is detected only when more energetic collisions are used to fragment *O*-acetylmandelate (Figure 4C) and acetoxyacetate (Figure 2B).

### Acetylsalicylate, 3a

**Figure 5.**
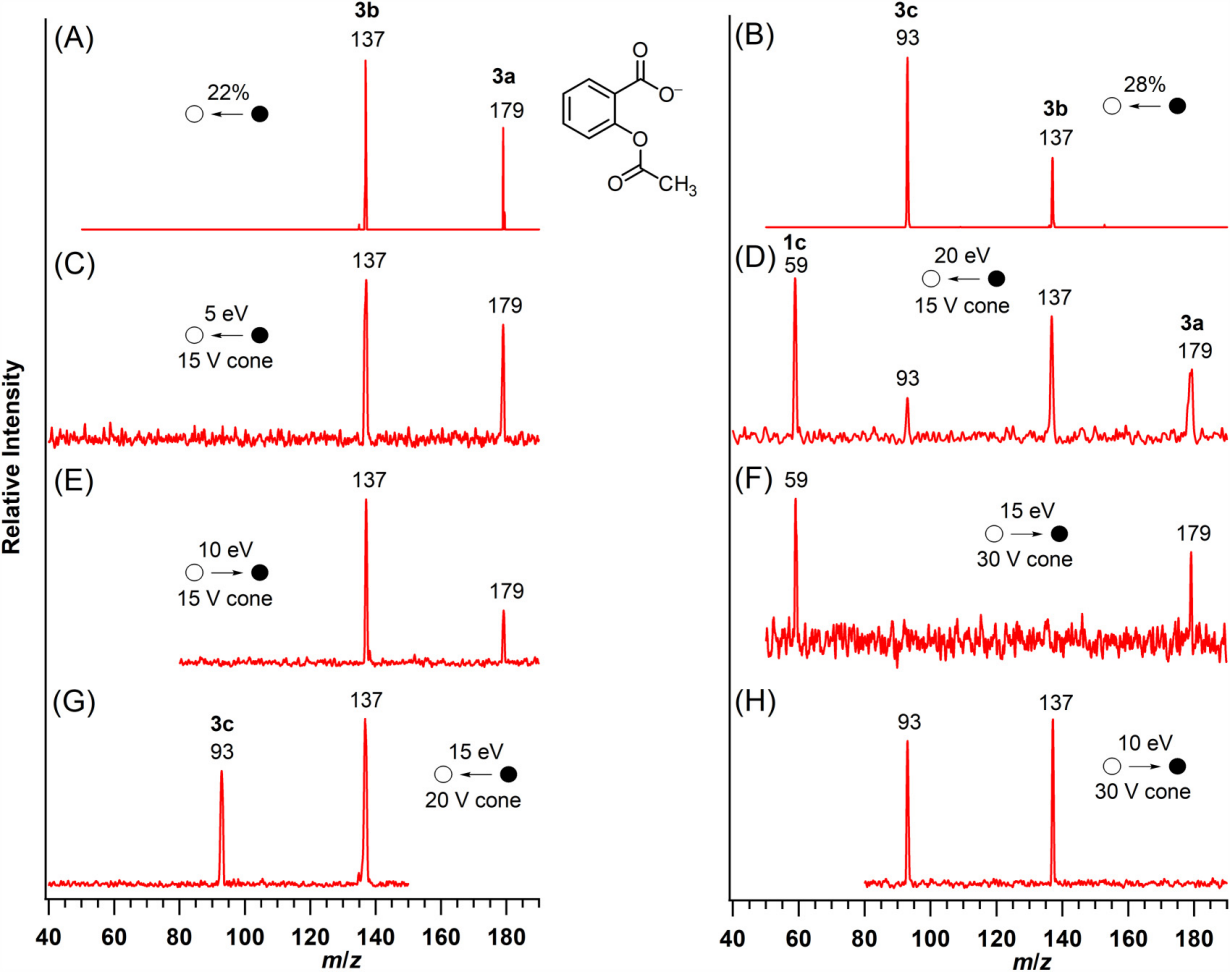
Mass spectra of acetylsalicylate (**3a**). A. MS/MS of **3a**; B. Pseudo MS^3^ of the *m*/*z* 137 ion; C. MS/MS of **3a** at low collision energy; D. MS/MS of **3a** at high collision energy; E. precursor-ion spectrum of the ion at *m*/*z* 137; F. Precursor-ion spectrum of the ion at *m*/*z* 59, **1c**; G. Pseudo MS^3^ of the *m*/*z* 137 ion; and H. Precursor-ion spectrum of the ion at *m*/*z* 93. Spectra A and B were collected on the ion-trap mass spectrometer and spectra C–H were collected on the triple quadrupole mass spectrometer.

The tandem mass spectra of acetylsalicylate (**3a**, *m*/*z* 179; Figure 5, Spectra A and C) showed the formation of a product ion at *m*/*z* 137 (**3b**) by the demonstrated neutral loss of ketene (42 u).^18^ When a higher collision energy was applied in the triple quadrupole mass spectrometer, a product ion at *m*/*z* 59 was also formed from acetylsalicylate (Figure 5D). The pseudo MS^3^ spectrum of the ion at *m*/*z* 137 (Figure 5, Spectra B and G) and a precursor-ion scan (Figure 5F) demonstrated that the product ions at *m*/*z* 137 (**3b**) and 59 (**3c**) were formed from acetylsalicylate (**3a**) by independent, competing processes. CID of **3b** (*m*/*z* 137) also yielded an ion at *m*/*z* 93 (**3d**) by decarboxylation (Figure 5, Spectra B and G), a common process documented for deprotonated aryl carboxylic acids.^1–4^ The pseudo MS^3^ spectra of **3b** (*m*/*z* 137; Figure 5, Spectra B and G) matched that of deprotonated salicylic acid,^31^ the structure ascribed to the *m*/*z* 137 ion formed from acetylsalicylate (**3a**, *m*/*z* 179) by loss of ketene.^18^ Moreover, the *m*/*z* 137 → 93 transition has been utilized in analytical determinations of salicylate.^13–17,32^

**Scheme 3.**
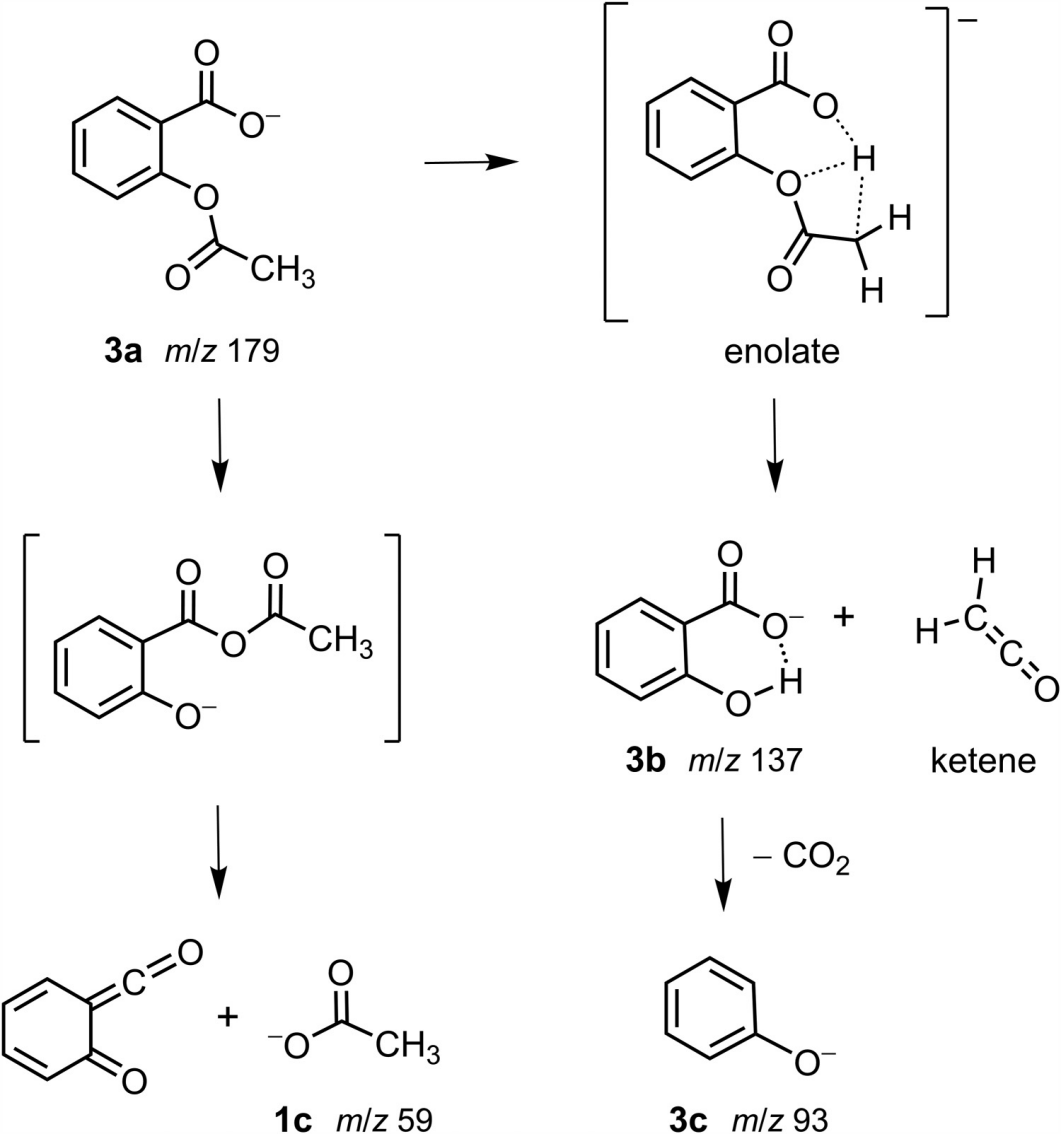
Competing fragmentation processes of acetylsalicylate (**3a**).

As characterized for acetoxyacetate (**1a**), loss of ketene from acetylsalicylate (**3a**) requires abstraction of a proton from the methyl group. The proximal positioning of the carboxylate and acetoxy groups on the aromatic ring and the rigid, planar alignment of four atoms in the eight-membered, cyclic transition state facilitate a proton transfer from the methyl group to a carboxylate oxygen atom (Scheme 3). Subsequent loss of ketene from the enolate generates salicylate (**3b**, *m*/*z* 137), an ion stabilized by internal H bonding. Loss of carbon dioxide and facile proton abstraction^4^ yields phenoxide as the ion observed at *m*/*z* 93 (**3c**).

At higher collision energy (Figure 5D), acetate ion (**1c**, *m*/*z* 59) is formed from acetylsalicylate (**3a**) by a competing fragmentation process (Scheme 3). Instead of an unfavorable nucleophilic substitution reaction on the aromatic ring, a likely reaction pathway is formation of an intermediate anhydride by acetyl group migration initiated by nucleophilic attack of the carboxylate oxygen on the carbonyl group of the ester. Subsequent heterolytic bond cleavage forms acetate (**1c**) and a neutral fragment containing the initial aryl ring.

## Conclusion

The *O*-acetyl-substituted carboxylate ions readily fragmented upon CID. For both acetoxyacetate and acetylsalicylate, two competing pathways were associated with bond cleavages in the *O*-acetyl group forming either a neutral product (ketene) or an ion (acetate) that retained the structural connections of the *O*-acetyl group (Schemes 1 and 3). In the lower energy pathway, the abstraction of a proton from the methyl group by the basic carboxylate group led to the formation of ketene (loss of 42 u). When each carboxylate ion was subjected to more energetic collisions, acetate ion (*m*/*z* 59) was produced. For acetoxyacetate, acetate was formed by an intramolecular nucleophilic displacement. The energy input computed for acetate displacement was only about 30% higher than that computed for ketene formation. However, the *O*-acetyl group in acetylsalicylate is bonded to the aryl ring, and acetate ion formation was proposed to occur after the rearrangement to an intermediate anhydride (Scheme 3).

Unlike acetoxyacetate and acetylsalicylate, the *O*-acetylmandelate ion decarboxylated upon CID demonstrating the structural dependance of fragmentation processes and the significant charge stabilizing effect of a phenyl substituent. Subsequent fragmentations of the decarboxylated ion were consistent with bond cleavages at the *O*-acetyl group leading to two pairs of complementary product ions (Scheme 2).

Overall, the reactions characterized in this study showed the carboxylate group participating as a base, acting as a nucleophile, or undergoing decarboxylation. Which process (or processes) is observed depends on the structures of the ions and the influence of intramolecular interactions, providing yet another example of the importance of these factors for the interpretation and prediction of the tandem mass spectra of multifunctional ions.

## Supplemental Material

sj-docx-1-ems-10.1177_14690667251346668 - Supplemental material for Competing fragmentation processes of *O*-acetyl-substituted carboxylate anions subjected to collision-induced dissociation Supplemental material, sj-docx-1-ems-10.1177_14690667251346668 for Competing fragmentation processes of *O*-acetyl-substituted carboxylate anions subjected to collision-induced dissociation by J Stuart Grossert and Robert L White in European Journal of Mass Spectrometry
