# Supplementary material for: Competing fragmentation processes of O-acetyl-substituted carboxylate anions subjected to collision-induced dissociation
Source: Eur J Mass Spectrom (Chichester). 2025 May 29;31(3-4):124–32. doi: 10.1177/14690667251346668 (PMC12314208; doi:10.1177/14690667251346668)
Supplement: sj-docx-1-ems-10.1177_14690667251346668 - Supplemental material for Competing fragmentation processes of O-acetyl-substituted carboxylate anions subjected to collision-induced dissociation [file sj-docx-1-ems-10.1177_14690667251346668.docx]

**Competing Fragmentation Processes of *O*-Acetyl Substituted Carboxylate Anions Subjected to Collision-Induced Dissociation**

J. Stuart Grossert and Robert L. White*

Department of Chemistry, Dalhousie University,

6243 Alumni Crescent, PO Box 15000, Halifax, Nova Scotia, B3H 4R2 Canada

*Correspondence: Email: robert.white@dal.ca

**Supplemental Material**

Atomic coordinates of computed structures, saddle points and ion-neutral complexes for product ions (MS^2^) in Figure 2. Computed energies are given in Hartree units as the “Sum of electronic and thermal Free Energies, EE+G”; energies relative to **1a** are reported in kJ mol^‒1^.

**1a**, ΔG = 0 kJ mol^‒1^, -456.406574 H

8 -0.571167000 -0.267357000 0.860956000

8 -1.459966000 -0.860623000 -1.136917000

6 -1.489976000 -0.225956000 -0.101979000

6 -2.609447000 0.724587000 0.265071000

1 -2.254901000 1.744338000 0.079978000

1 -3.486199000 0.525516000 -0.353715000

1 -2.859329000 0.646606000 1.326966000

6 0.735832000 -0.813729000 0.539894000

1 1.126375000 -1.184688000 1.488748000

1 0.619504000 -1.641551000 -0.162135000

6 1.715907000 0.248856000 -0.054695000

8 1.235404000 1.353963000 -0.375862000

8 2.888311000 -0.187580000 -0.131877000

**TS1a‒enol**, ΔG = +102 kJ mol^‒1^, -456.373020 H

8 -0.623866000 -0.748330000 0.886862000

8 -2.016436000 -0.764080000 -0.923291000

6 -1.550694000 -0.112712000 0.002695000

6 -1.618747000 1.279240000 0.282703000

1 -0.157360000 1.376058000 -0.054271000

1 -2.358959000 1.843887000 -0.282137000

1 -1.514064000 1.558903000 1.330230000

6 0.604449000 -1.075249000 0.252166000

1 1.155269000 -1.727842000 0.933080000

1 0.419519000 -1.629695000 -0.678774000

6 1.528660000 0.116494000 -0.085593000

8 1.005127000 1.304847000 -0.196619000

8 2.719374000 -0.125930000 -0.261947000

**Enolate**, ΔG = +89 kJ mol^‒1^, --456.372856 H

8 -0.569974000 -0.593091000 0.942228000

8 -1.808332000 -0.893692000 -0.980189000

6 -1.564150000 -0.137570000 -0.032178000

6 -1.976681000 1.153077000 0.266738000

1 0.015890000 1.323075000 -0.135055000

1 -2.778055000 1.591616000 -0.318361000

1 -1.753694000 1.574828000 1.240417000

6 0.622886000 -1.024509000 0.342589000

1 1.172346000 -1.644303000 1.056988000

1 0.414130000 -1.626878000 -0.553566000

6 1.558913000 0.117858000 -0.073090000

8 1.013720000 1.318979000 -0.266002000

8 2.750032000 -0.066131000 -0.235384000

**TSenol‒1b**, ΔG = +108 kJ mol^‒1^, -456.374473 H

8 -0.355544000 0.607596000 0.565526000

8 4.319293000 -0.940893000 -0.297042000

6 3.554326000 -0.067078000 -0.129698000

6 2.699498000 0.903538000 0.059013000

1 -0.942367000 1.254752000 0.093824000

1 3.064422000 1.923283000 0.066158000

1 1.629827000 0.699802000 0.204575000

6 -1.217406000 -0.529424000 0.550035000

1 -0.751016000 -1.359254000 0.001006000

1 -1.417937000 -0.868536000 1.574996000

6 -2.559221000 -0.150433000 -0.141029000

8 -2.591277000 1.048105000 -0.545727000

8 -3.428235000 -1.038515000 -0.219068000

**IN** , ΔG = +102 kJ mol^‒1^, -456.376576 H

8 -0.300908000 0.197573000 -0.905785000

8 4.537921000 -0.233175000 -0.236580000

6 3.486710000 0.005539000 0.228473000

6 2.312402000 0.270269000 0.736481000

1 -0.820131000 1.027922000 -0.739936000

1 2.237537000 0.509438000 1.790121000

1 1.408688000 0.250761000 0.108082000

6 -1.239070000 -0.755790000 -0.406212000

1 -1.531579000 -1.453961000 -1.201788000

1 -0.792705000 -1.339702000 0.410598000

6 -2.500773000 -0.013248000 0.120287000

8 -2.427901000 1.244027000 -0.004549000

8 -3.416291000 -0.712810000 0.591758000

**Ketene**, -152.610803 H

8 -1.267377000 -0.000001000 0.000001000

6 -0.102467000 0.000017000 -0.000007000

6 1.210759000 0.000010000 0.000015000

1 1.744739000 0.940463000 -0.000029000

1 1.744528000 -0.940617000 -0.000029000

**1b, m/z75**, ΔG = +102 kJ mol^‒1^, -303.767922 H

8 -1.806776000 0.052987000 0.000129000

1 -1.363825000 0.934262000 0.000097000

6 -0.661646000 -0.791391000 -0.000125000

1 -0.663413000 -1.440290000 0.887825000

1 -0.663486000 -1.439887000 -0.888377000

6 0.641352000 0.058194000 -0.000009000

8 0.436138000 1.305724000 -0.000070000

8 1.722199000 -0.565574000 0.000098000

**TS1a‒1c**, ΔG = +135 kJ mol^‒1^, -456.362087 H

8 1.636048000 1.072160000 -0.661788000

8 1.182801000 -0.671070000 0.696515000

6 1.948646000 0.046369000 -0.004800000

6 3.424774000 -0.408890000 -0.083091000

1 3.732721000 -0.895602000 0.848033000

1 3.522106000 -1.144705000 -0.891939000

1 4.083632000 0.434943000 -0.310872000

6 -1.142025000 0.433726000 0.447779000

1 -0.927082000 0.347430000 1.503205000

1 -0.423991000 0.961047000 -0.176986000

6 -2.300971000 -0.235686000 -0.099565000

8 -2.809944000 -1.212065000 -0.581603000

8 -2.705147000 0.971447000 0.230203000

**IN1c‒AL,** ΔG = +126 kJ mol^‒1^, -456.365165 H

8 1.539438000 0.946051000 -0.656091000

8 1.450999000 -0.850767000 0.706883000

6 2.050338000 -0.012751000 -0.014967000

6 3.583437000 -0.177030000 -0.156557000

1 3.980857000 -0.868884000 0.592291000

1 3.807878000 -0.572086000 -1.155768000

1 4.079559000 0.796600000 -0.074435000

6 -1.204887000 0.218429000 0.458855000

1 -0.937929000 -0.112041000 1.455291000

1 -0.410944000 0.700872000 -0.119619000

6 -2.463195000 -0.190271000 -0.117327000

8 -3.163296000 -0.962900000 -0.711497000

8 -2.616338000 0.995775000 0.445982000

**AL, acetolactone**, -227.816270 H

6 1.027171000 -0.593655000 -0.000009000

1 1.502082000 -0.900009000 0.927162000

1 1.501697000 -0.900559000 -0.927209000

6 -0.325263000 -0.068987000 0.000179000

8 -1.509273000 -0.160627000 -0.000042000

8 0.607369000 0.882679000 -0.000080000

**1c, m/z59**, ΔG = +174 kJ mol^‒1^, -228.533653 H

8 0.750539000 1.134374000 0.001809000

8 0.756213000 -1.131480000 0.001796000

6 0.209290000 0.000061000 -0.009172000

6 -1.352102000 -0.002809000 -0.005032000

1 -1.746861000 -0.912604000 -0.471609000

1 -1.701845000 0.019514000 1.036635000

1 -1.748439000 0.886424000 -0.508638000
